# Is patient satisfaction with organizational aspects of their general practitioner’s practice associated with patient and doctor gender? An observational study

**DOI:** 10.1186/s12875-016-0513-0

**Published:** 2016-08-27

**Authors:** Paul Sebo, François R. Herrmann, Dagmar M. Haller

**Affiliations:** 1 Primary Care Unit, Faculty of medicine, University of Geneva, Geneva, Switzerland; 2 Geriatrics Division, Department of Internal Medicine, Rehabilitation and Geriatrics, Geneva University Hospitals, Geneva, Switzerland; 3 Department of Community, Primary Care and Emergency Medicine, Geneva University Hospitals, Geneva, Switzerland; 4 Department of Pediatrics, Geneva University Hospitals, Geneva, Switzerland; 5 Centre médico-chirurgical des Trois Chêne, 97-99, rue de Genève, 1226 Thônex, GE Switzerland

**Keywords:** Assessment, Patient satisfaction, Gender, Primary care

## Abstract

**Background:**

No study has assessed the association between patients’ and doctors’ gender and patient satisfaction with organizational aspects of health care in primary care. However, just like satisfaction regarding communication styles or technical skills, satisfaction towards organization of the general practitioner (GP) practice could also depend on doctors’ and/or patients’ gender. Different expectations between female and male patients regarding the organization of the practice or different ways of organizing care delivery between female and male GPs could act on this satisfaction. We aimed to compare female and male patients’ satisfaction towards their GP overall, and according to GPs’ gender.

**Methods:**

In a cross-sectional study in Geneva, 23 randomly selected GPs (participation rate: 31 %) were asked to recruit up to 100 consecutive patients coming to the practice for a scheduled medical consultation. The patients completed an anonymous questionnaire about their satisfaction with their GP. Patient satisfaction was assessed using the six questions from the Europep questionnaire regarding organizational aspects of health care in terms of accessibility and availability, and presented in two different ways: % of patients very satisfied and mean score (SD). Multivariate analyses adjusting for patient and GP characteristics were conducted to compare outcomes between genders.

**Results:**

One thousand six hundred thirty-seven patients agreed to participate (participation rate: 97 %, women: 63 %, mean age: 54 years). The majority of patients were very satisfied (women 96.2 %, men 95.3 %, *p* = 0.38). Mean satisfaction scores were slightly higher in women (for overall satisfaction: women 4.7/5 (SD 0.6), men 4.6/5 (SD 0.6), *p* = 0.02) and in women visiting male GPs (women 4.6 (SD 0.6), men 4.5 (SD 0.6), *p* = 0.01), and the gender differences showed consistency across satisfaction items. These differences were small and no longer statistically significant in multivariate analyses.

**Conclusions:**

These findings suggest that patients are highly satisfied with the organization of their GP’s practice, regardless of patients’ and GPs’ gender. As patients’ and GPs’ gender are known to influence patient satisfaction towards primary care delivery and as the current study is the first to explore this aspect in relation to organizational aspects of GP practice, further studies are needed in various primary care settings to confirm our results.

## Background

Patient satisfaction is an important indicator of health quality [1, 2]. As it may influence not only patients’ health status but also medical costs it is considered a key factor in the assessment of health care services [3]. Many authors have in particular investigated patients’ satisfaction with the quality of communication between health care providers and their patients, and it has been shown that various aspects of verbal and nonverbal communication can affect patients’ satisfaction and their likelihood to adhere to general practitioners’ (GPs) recommendations and to return to their GP for subsequent consultations [4, 5].

Several authors have assessed patients’ satisfaction with medical health care services in primary care, in particular in the context of the Europep project (EUROpean task force on Patient Evaluation of general Practice), using an internationally developed instrument to evaluate general practice care from the perspective of patients. These studies consistently showed a high degree of satisfaction among patients [6–10].

Some studies have also assessed the influence of patients’ and/or doctors’ gender on satisfaction ratings in various contexts [11, 12]. Interestingly, a mailed survey carried out in a group-model HMO setting in northern California (USA) in 1995–96 which assessed various communication and technical skills showed that patients having chosen a GP of the opposite gender tended to be more satisfied in general than those having chosen a GP of the same gender [12]. Though the authors of this study did not provide conclusive explanations for these findings, differences in patient satisfaction may be explained by different styles of care of male and female doctors and different expectations of male and female patients towards their GP [4, 12–16]. Male and female GPs manage different types of medical conditions [12, 17–20]. Female GPs tend to manage more female-specific and psychosocial problems, and they seem to communicate differently with their patients than their male counterparts, discussing social concerns and using shared decision-making style more often [4, 12]. They also seem to spend more time with their patients than males [4, 12]. Furthermore, female patients’ expectations seem to be higher regarding some issues, such as social problems, lifestyle and prevention, than their male counterparts [12].

Communication and technical skills are widely taught during medical studies and postgraduate training, unlike organization of care. However, patients have a wide range of expectations regarding organizational aspects of care which should be taken into account when considering potential improvements to quality of primary care [21]. To our knowledge, no study has evaluated the influence of gender on satisfaction with organizational aspects of health care in primary care, such as availability and accessibility. Yet, just like gender differences in satisfaction regarding communication styles or technical skills, satisfaction towards organization of the GP practice could also depend on doctors’ and patients’ gender. These differences could reflect different expectations between female and male patients regarding the organization of their GP’ practice or different ways of organizing care delivery between female and male GPs. Assessing these potential differences could benefit to both, GPs and their patients, as GPs could adjust their practice organizations to patients’ gender to better meet their expectations. This could be particularly important for GPs whose patient panel is biased towards one or the other gender, who could adapt the organization of their practice to gender-related trends. It could help GPs meet their patients’ expectations with positive effects on patient-doctor relationships, compliance and, consequently, health status. For example, the identification that accessing the practice on the phone is essential for women could lead GPs who have a large panel of female patients to prioritize telephone access over other organizational aspects in their practice.

Therefore, as part of a larger study having explored patient satisfaction with organizational aspects of health care in primary care, we aimed to compare female and male patients’ satisfaction with the organization of their doctor’s practice in terms of accessibility and availability, first overall, then according to GPs’ gender.

## Methods

This cross-sectional survey was carried out in the Geneva area, Switzerland, in 2011. A sample of 75 GPs was randomly selected, without exclusion criteria, from the list of all GPs practising in the Geneva area and invited to participate by post, in order to include 25 GPs in the study. The GPs who did not respond to the invitation were contacted by phone two or three weeks later. A research assistant contacted each participating GP’s medical assistant to inform them about the practical procedures for data collection. The GPs were asked to recruit between 50 and 100 consecutive patients coming to the practice for a planned consultation. The patients were given oral and written information and, following written consent, were asked to complete an anonymous questionnaire containing general questions (gender, age, nationality, marital status, completed training, work status and health status) and questions about their satisfaction with the organization of their GP’s practice.

Patient satisfaction was assessed using six questions from the Europep standardized questionnaire, validated in French, and scored on a 5 point Likert scale ranging from “poor” to “excellent”: helpfulness of staff, getting an appointment to suit the patient, getting through to the practice on the telephone, being able to speak to the doctor on the telephone, waiting time in the waiting room, and providing quick services for urgent health problems [6, 22, 23]. We included in the questionnaire only these six items evaluating accessibility and availability, because we were interested in assessing organizational aspects of health care. Note that we used the Europep questionnaire (EURopean task force on Patient Evaluation of general practice) for several reasons: the instrument was recently developed in order to assess patient satisfaction in primary care, many authors have used this questionnaire to assess patient satisfaction with medical health care delivery in primary care, the questionnaire is standardised, validated in French and easy to use, the evaluation is based on patients’ priorities, and organization of the GP practice is one of the five dimensions assessed by this instrument. When comparing validated instruments evaluating satisfaction towards organization of practices in primary care (Primary Care Assessment Survey (PCAS), Primary Care Assessment Tool (PCAT) and Europep questionnaire), the Europep questionnaire demonstrates excellent performance for measuring organization of care delivery in terms of accessibility and availability [24].

The questionnaire was pretested in a GP practice (PS) and feedback was obtained from respondents (*n* = 20), in order to identify any difficulties patients could meet in responding to the questions. Eligibility criteria were age > 15 years, ability to understand and write French, having a planned consultation with the doctor and informed consent. All new patients and those who were seen for an emergency were excluded. The self-administered anonymous questionnaire had to be completed in the waiting room of the practice, before or after the consultation, and deposited at the desk in a closed box. The study was approved by the local ethical research committee under reference number 09/01.

The sample size was estimated in order to measure percentages for categorical data with a margin of error inferior to 5 %. Taking the cluster effect into account, with an intra-class correlation of 0.025 (estimate based on published data and our personal experience) [25], our estimated total sample size was 1392 patients. We computed the percentage of patients being satisfied or very satisfied (4 or 5 /5 on the Likert satisfaction scale), as well as the mean score. For categorical data, we used Chi-squared tests to compare the percentages obtained in men and women, and conditional logistic regression to simultaneously adjust for doctor (gender, age, certification, number of employees in the practice, location and type of practice, number of days worked per week and number of working-years since certification) and patient (age, nationality, marital status, completed training, work status and health status) characteristics, whereas for continuous data, we used analysis of variance and multiple linear regression with adjustment for the same variables as described above. Analysis of variance, logistic and linear regression were performed while taking into account the clustering of the observations (the fact that many patients consulted the same GP).

## Results

Twenty-three out of 75 GPs, practicing in 23 different practices, agreed to participate (31 %). Table 1 presents the GPs’ characteristics. Their mean age was 50 years (min-max: 35–65 years). They were predominantly male (61 %), practicing in an urban setting (52 %), in solo (39 %) or duo (35 %) practices, and were relatively experienced doctors (average years worked as GP: 10.5 (SD 10.1)). On average, they were working 4.7 days per week. They seemed to be representative of the GPs practising in the Geneva area (*n* = 650, mean age: 53 years, men: 61 %).

**Table 1:** GPs’ socio-demographic characteristics (*n* = 23)

Characteristics	% or mean (SD)	Min-max
Male	60.9	
Mean age (SD)	47.9 (9.2)	35–65
Urban practice (area with > 15’000 inhabitants)	52.2	
Number of doctors practicing in the practice		
1	39.1	
2	34.8	
3	4.4	
≥ 4	21.7	
Number of staff working in the practice		
0	4.4	
1	39.1	
2	21.7	
3	13.0	
≥ 4	21.7	
Mean number of days worked per week (SD)	4.7 (0.6)	3.5–5.5
Mean number of years in the current practice (SD)	8.6 (8.6)	1.5–31
Mean number of working-years as GP (SD)	10.5 (10.1)	1.5–31

One thousand six hundred thirty-seven patients agreed to participate in the study (approximately 250 above the expected sample size). Only 45 patients declined participation (participation rate > 97 %). Participants were predominantly women (63 %), aged 54 years on average (SD 18). Half the patients were married, and three quarters were Swiss. Almost one third completed a university training or equivalent, and more than half had education beyond primary school. The majority had a professional activity (41 %) or was retired (30 %). On average, they were in good health; only 18 % rated their health as moderate or poor.

Table 2 shows the satisfaction levels according to patients’ gender, presented in two different ways: the % of patients very satisfied (i.e. rated 4/5 or 5/5) and the mean score (SD). Overall, the vast majority of the patients were very satisfied, mainly concerning the overall satisfaction and the helpfulness of staff with ≥ 95 % of patients being very satisfied (mean scores ≥ 4.6), though two items, the possibility to speak to the doctor by phone and the waiting time in the waiting room, were rated less favorably by the patients. The satisfaction levels tended to be slightly higher in women, and the differences were statistically significant for four items (overall satisfaction (women 4.7 (SD 0.6), men 4.6 (SD 0.6), *p* = 0.02), getting an appointment to suit the patient (4.6 (SD 0.7) vs. 4.5 (SD 0.7), *p* = 0.02), being able to speak to the GP on the telephone (4.3 (0.9) vs. 4.2 (1.0), *p* = 0.03) and providing quick services for urgent health problems (4.5 (SD 0.7) vs. 4.4 (SD 0.8), *p* = 0.02) when satisfaction levels were reported as mean scores, but not when they were reported as % of patients who were very satisfied. After having stratified the data into two groups according to GPs’ gender (see Tables 3 and 4), women remained slightly more satisfied than men towards male GPs, and all the differences were statistically significant when using mean scores, but not when using the % of patients who were very satisfied. Alternatively, men tended to be more satisfied than women towards female GPs, though the differences, reported as either mean scores or % of patients very satisfied, were not statistically significant. All the differences presented in Tables 2, 3 and 4 were no longer statistically significant when controlling for patient and doctor characteristics, including patients’ and GPs’ age.

**Table 2:** Patients’ satisfaction levels with GPs and their practices, according to patients’ gender (crude and adjusted *p*-values relate to the significance of the difference in satisfaction for women compared with men)

Characteristics	Women	Men	Crude *p*-value	Adjusted *p*-value^a^
Overall satisfaction level				
Very high to excellent satisfaction level, %	96.2	95.3	0.38	0.95
Mean satisfaction score (SD)	4.7 (0.6)	4.6 (0.6)	0.02	0.36
Helpfulness of the staff (other than the GP)				
Very high to excellent satisfaction level, %	94.6	95.5	0.46	0.30
Mean satisfaction score (SD)	4.7 (0.6)	4.7 (0.6)	0.09	0.53
Getting an appointment to suit the patient				
Very high to excellent satisfaction level, %	92.3	91.6	0.66	0.75
Mean satisfaction score (SD)	4.6 (0.7)	4.5 (0.7)	0.02	0.37
Getting through to the practice on the telephone				
Very high to excellent satisfaction level, %	90.6	89.5	0.52	0.68
Mean satisfaction score (SD)	4.5 (0.8)	4.4 (0.7)	0.07	0.23
Being able to speak to the PCP on the telephone				
Very high to excellent satisfaction level, %	82.6	79.2	0.11	0.63
Mean satisfaction score (SD)	4.3 (0.9)	4.2 (1.0)	0.03	0.20
Waiting time in the waiting room				
Very high to excellent satisfaction level, %	76.7	74.0	0.24	0.74
Mean satisfaction score (SD)	4.0 (1.0)	4.0 (0.9)	0.14	0.68
Providing quick services for urgent health problems				
Very high to excellent satisfaction level, %	91.4	88.5	0.08	0.67
Mean satisfaction score (SD)	4.5 (0.7)	4.4 (0.8)	0.02	0.38

^a^adjusted for patient characteristics (age, nationality, marital status, completed training, work status and health status) and doctor characteristics (gender, age, certification, number of employees in the practice, location and type of practice, number of days worked per week and number of working-years since certification)

**Table 3:** Patients’ satisfaction levels with male GPs and their practices, according to patients’ gender (crude and adjusted *p*-values relate to the significance of the difference in satisfaction for women compared with men)

Characteristics	Women	Men	Crude *p*-value	Adjusted *p*-value^a^
Overall satisfaction level				
Very high to excellent satisfaction level, %	96.0	93.9	0.16	0.68
Mean satisfaction score (SD)	4.6 (0.6)	4.5 (0.6)	0.01	0.31
Helpfulness of the staff (other than the GP)				
Very high to excellent satisfaction level, %	94.3	94.0	0.85	0.67
Mean satisfaction score (SD)	4.7 (0.6)	4.6 (0.7)	0.03	0.64
Getting an appointment to suit the patient				
Very high to excellent satisfaction level, %	92.5	90.8	0.36	0.69
Mean satisfaction score (SD)	4.6 (0.7)	4.4 (0.7)	0.004	0.54
Getting through to the practice on the telephone				
Very high to excellent satisfaction level, %	90.7	86.4	0.05	0.18
Mean satisfaction score (SD)	4.5 (0.8)	4.4 (0.8)	0.01	0.14
Being able to speak to the PCP on the telephone				
Very high to excellent satisfaction level, %	79.5	73.3	0.04	0.42
Mean satisfaction score (SD)	4.2 (1.0)	4.0 (1.0)	0.01	0.19
Waiting time in the waiting room				
Very high to excellent satisfaction level, %	76.1	68.5	0.01	0.21
Mean satisfaction score (SD)	4.0 (1.0)	3.8 (0.9)	0.01	0.36
Providing quick services for urgent health problems				
Very high to excellent satisfaction level, %	90.2	86.1	0.07	0.96
Mean satisfaction score (SD)	4.5 (0.8)	4.4 (0.8)	0.03	0.78

^a^adjusted for patient characteristics (age, nationality, marital status, completed training, work status and health status) and doctor characteristics (age, certification, number of employees in the practice, location and type of practice, number of days worked per week and number of working-years since certification)

**Table 4:** Patients’ satisfaction levels with female GPs and their practices, according to patients’ gender (crude and adjusted *p*-values relate to the significance of the difference in satisfaction for women compared with men)

Characteristics	Women	Men	Crude *p*-value	Adjusted *p*-value^a^
Overall satisfaction level				
Very high to excellent satisfaction level, %	96.5	97.4	0.56	0.76
Mean satisfaction score (SD)	4.7 (0.5)	4.7 (0.5)	0.82	0.88
Helpfulness of the staff (other than the GP)				
Very high to excellent satisfaction level, %	95.1	97.8	0.10	0.13
Mean satisfaction score (SD)	4.7 (0.6)	4.8 (0.5)	0.79	0.99
Getting an appointment to suit the patient				
Very high to excellent satisfaction level, %	91.9	92.9	0.65	0.86
Mean satisfaction score (SD)	4.6 (0.7)	4.6 (0.6)	0.92	0.83
Getting through to the practice on the telephone				
Very high to excellent satisfaction level, %	90.4	94.3	0.09	0.28
Mean satisfaction score (SD)	4.5 (0.8)	4.5 (0.6)	0.59	0.96
Being able to speak to the PCP on the telephone				
Very high to excellent satisfaction level, %	86.7	87.9	0.69	0.99
Mean satisfaction score (SD)	4.3 (0.9)	4.3 (0.8)	0.96	0.57
Waiting time in the waiting room				
Very high to excellent satisfaction level, %	77.6	82.6	0.14	0.52
Mean satisfaction score (SD)	4.0 (0.9)	4.1 (0.8)	0.16	0.64
Providing quick services for urgent health problems				
Very high to excellent satisfaction level, %	93.0	92.5	0.82	0.57
Mean satisfaction score (SD)	4.6 (0.7)	4.6 (0.7)	0.42	0.56

^a^adjusted for patient characteristics (age, nationality, marital status, completed training, work status and health status) and doctor characteristics (age, certification, number of employees in the practice, location and type of practice, number of days worked per week and number of working-years since certification)

## Discussion

Overall, we found high satisfaction levels with some organizational aspects of care (accessibility and availability), though two items (the possibility to speak to the GP by phone and the waiting time in the waiting room) were slightly less well rated. The satisfaction levels tended to be higher in women overall and in women visiting male GPs, though the differences were not statistically significant in multivariate analysis, and so small that they are unlikely to be truly significant in practice.

The finding that the vast majority of the 1637 patients who participated in the study reported high satisfaction with organizational aspects of care is not new and was discussed in our previous paper which compared our findings with those coming from studies having used the same questionnaire (Europep) [21].

We showed that the satisfaction levels tended to be slightly higher in women overall and in women visiting male GPs, and the differences showed consistency across satisfaction items, suggesting that female and male patients may have different expectations regarding practice organization, though the differences were unlikely to be clinically relevant (0.1–0.2 point difference for the mean scores) and not statistically significant when adjusting for a certain number of doctor and patient socio-demographic characteristics. Literature on this topic is extremely scarce to our knowledge, but these findings are in line with the relatively old study carried out in a group-model HMO setting in California (USA) in 1995–96 (*n* = 10’205) which assessed various communication and technical skills, and showed that patients having chosen a GP of the opposite gender tended to be more satisfied in general than those having chosen a GP of the same gender [12]. The association between gender and responses to the Europep questionnaire were previously briefly discussed by several authors. Gender data were not, however, available in these papers and the questionnaire was not restricted to organizational aspects of care. These rather old studies carried out in ten European countries (*n* = 17’391) [6], respectively in Belgium (*n* = 994) [10] and in Slovenia (*n* = 1’812) [8] did not show any association between patients’ and/or GPs’ gender and satisfaction.

Though the gender differences found in our study were small, we consider three possible explanations for gender differences in satisfaction. First, female patients could have a tendency to be more critical towards GPs of the same gender, regardless of their expectations, which is not necessary the case for male patients visiting male GPs. Second, female GPs tend to spend more time in consultation than males [4, 12], and could be therefore less available for other patients, for instance those asking to speak to the doctor on the telephone or asking for urgent health problems; In other words, trying to satisfy patients within the consultation, female GPs take the risk to dissatisfy other patients, particularly under time pressure. Alternatively, male GPs seem to have the tendency to reduce information exchange and to give shorter answers to patients’ questions than their female counterparts [4]. Although this style of communication increases the risk of dissatisfying patients, it has the advantage to limit consultation delays, which could explain why female patients tend to be more satisfied with male GPs in our study. The finding that male patients did not grade female GPs less than males could be explained by the fact that dissatisfaction regarding the practice’s organization could be minimized by higher satisfaction regarding other domains, mainly communication skills. Third, it was suggested that differences in patient satisfaction in general may be explained by different styles of care of male and female doctors and different expectations of male and female patients towards their GP [4, 12–16]; it has been shown that female doctors were more oriented than males toward communication issues, were more often discussing social concerns and using shared decision-making style [4, 12]. Yet, female patients having chosen female GPs, as they were looking for a certain style of medical care, could be particularly disappointed if they had relatively high expectations from their female GPs. In addition, though female GPs tend to spend more time than males in face-to-face interaction with patients, it has been suggested that, under time pressure, female GPs could need to reduce time spent with patients and be more often stressed that males, which could alter quality of the consultation [12]. These factors could contribute to decrease female patients’ satisfaction with their female GP’s communication skills; though they should not directly influence satisfaction regarding organizational aspects of care, it is possible that if some concerns were not adequately addressed during the consultation, female patients would have tendency to be more critical about organization of care of their female GP, thus underestimating their satisfaction level.

Though the number of patients who participated in our study was above the estimated sample size, the absence of statistically significant association between gender and satisfaction for the % of patients being very satisfied could be due to a type II error (i.e. the null hypothesis is not rejected when it is false), as results showed consistency across satisfaction items (similar results are seen for various satisfaction items) and statistically significant associations for the majority of the items of Tables 2 and 3 when considering the alternative way of presenting the results (mean satisfaction scores). However, even if all these differences were statistically significant, they would be unlikely to be clinically pertinent, as they were small.

Our work has several limitations. First, it was conducted in an urban region and thus may not be generalizable to more rural areas. Then, the patients having consulted in an emergency situation or who did not speak French were excluded from the study. These patients could have other views regarding satisfaction and are likely to have lower health and/or socio-economic status. Only 31 % of the GPs who were contacted agreed to participate, which may have introduced a selection bias, as these GPs could be more interested by the study topic and therefore by their patients’ satisfaction. Finally, gender was considered as a binary variable (female and male patients, female and male GPs); however, a wider variation of gender could have been considered, given the diversity of patients presenting in primary care settings; in particular, transgender patients could have other expectations regarding practice organization.

## Conclusion

These findings highlight the relatively high satisfaction levels with organizational aspects of GP care regarding accessibility and availability, and the absence of any statistical significant association between gender and satisfaction in multivariable analyses. As patients’ and GPs’ gender are known to influence patient satisfaction towards primary care delivery and as the current study is to our knowledge the first dedicated to organizational aspects of GP practice, further studies should be carried out in a variety of primary care settings to address this issue, in particular in primary care settings where gender is more likely to play an important role such as sexual health or family planning services.

## Abbreviations

Europep: European task force on patient evaluation of general practice
GP(s): General practitioner(s)
